# Whole blood viscosity is associated with baseline cerebral perfusion in acute ischemic stroke

**DOI:** 10.1007/s10072-021-05666-5

**Published:** 2021-10-20

**Authors:** Prajwal Gyawali, Thomas Patrick Lillicrap, Shinya Tomari, Andrew Bivard, Elizabeth Holliday, Mark Parsons, Christopher Levi, Carlos Garcia-Esperon, Neil Spratt

**Affiliations:** 1 Priority Research Centre for Stroke and Brain Injury, Hunter Medical Research Institute, New Lambton Heights, New South Wales Australia; 2 Faculty of Health, Engineering and Sciences, School of Health and Wellbeing, University of Southern Queensland, Toowoomba, Queensland Australia; 3 Department of Neurology, John Hunter Hospital, New Lambton Heights, New South Wales Australia; 4 Department of Neurology, Royal Melbourne Hospital, Parkville, Victoria Australia; 5 School of Medicine and Public Health, The University of Newcastle, Callaghan, New South Wales Australia; 6 University of New South Wales, Sydney, New South Wales Australia; 7 John Hunter Hospital, Hunter New England Health, New Lambton Heights, New South Wales Australia; 8 Education, Research and Enterprise, Sydney Partnership for Health, Liverpool, New South Wales Australia; 9 School of Biomedical Sciences and Pharmacy, The University of Newcastle, Callaghan, New South Wales Australia

**Keywords:** Stroke, Whole blood viscosity, CT perfusion

## Abstract

Whole blood viscosity (WBV) is the intrinsic resistance to flow developed due to the frictional force between adjacent layers of flowing blood. Elevated WBV is an independent risk factor for stroke. Poor microcirculation due to elevated WBV can prevent adequate perfusion of the brain and might act as an important secondary factor for hypoperfusion in acute ischaemic stroke. In the present study, we examined the association of WBV with basal cerebral perfusion assessed by CT perfusion in acute ischaemic stroke. Confirmed acute ischemic stroke patients (*n* = 82) presenting in hours were recruited from the single centre. Patients underwent baseline multimodal CT (non-contrast CT, CT angiography and CT perfusion). Where clinically warranted, patients also underwent follow-up DWI. WBV was measured in duplicate within 2 h after sampling from 5-mL EDTA blood sample. WBV was significantly correlated with CT perfusion parameters such as perfusion lesion volume, ischemic core volume and mismatch ratio; DWI volume and baseline NIHSS. In a multivariate linear regression model, WBV significantly predicted acute perfusion lesion volume, core volume and mismatch ratio after adjusting for the effect of occlusion site and collateral status. Association of WBV with hypoperfusion (increased perfusion lesion volume, ischaemic core volume and mismatch ratio) suggest the role of erythrocyte rheology in cerebral haemodynamic of acute ischemic stroke. The present findings open new possibilities for therapeutic strategies targeting erythrocyte rheology to improve cerebral microcirculation in stroke.

## Introduction

Whole blood viscosity (WBV) is the intrinsic resistance to flow developed due to the frictional force between adjacent layers of flowing blood. Elevated WBV reduces tissue perfusion in the affected organ [1–3]. For instance, it is associated with reduced coronary collateral circulation in patients with chronic total occlusion [4], and with increased myocardial infarct size and left ventricular dysfunction [2]. More recently, WBV is also shown to be an independent and significant predictor of early left ventricular apical thrombus formation complicating a first-ever anterior wall myocardial infarction [5]. In addition, elevated WBV is a risk factor of primary and secondary stroke [6–8], and possibly could be an important secondary factor determining cerebral reperfusion in acute ischemic stroke.

Cerebral blood vessels have an essential ability to auto-regulate their blood flow by altering their diameter maintaining perfusion with minimal changes to the flow dynamics. After an ischemic stroke however, the affected blood vessels exhaust their auto-regulatory capacity [9]. The reperfusion therapies, thrombolysis and endovascular thrombectomy, pursue vessel recanalization, but the time between stroke onset and recanalization can be delayed for many hours. This leads to progression of tissue infarction (core) and decreases the volume of salvageable tissue (penumbra). The rate at which the infarct grows varies widely [10, 11], and depends on several secondary variables such as collateral circulation [12, 13] and competent cerebral microcirculation [14].

Poor microcirculation due to elevated WBV can prevent adequate perfusion of the brain at the advent of failed auto-regulation and might act as an important secondary factor for hypoperfused tissue growth. In cerebral microvasculature, erythrocytes must traverse through blood vessel whose diameter is much smaller than their own (typically 7 µm). Therefore, to facilitate tissue perfusion, erythrocytes need to be able to deform and assume a parachute-like shape. In the state of elevated WBV, which involves both increased plasma viscosity and decreased erythrocyte deformability, this physiological process is inhibited leading to increased stenotic peripheral resistance and microcirculation sludging [15].

The definitive role of WBV in determining the perfusion lesion and hypoperfused tissue growth in acute ischemic stroke is unknown, but previous studies have suggested a role for elevated WBV in influencing cerebral perfusion, in general [16–18]. While these previous studies [16–19] do provide proof-of-concept regarding the reciprocal association of WBV and cerebral blood flow, they either did not use contemporary technique to quantify cerebral perfusion or did not measure the perfusion specifically in hypoperfused regions of the brain. In the present study, we aim to examine the association between WBV and cerebral perfusion in a cohort of confirmed acute ischemic stroke patients who underwent baseline multimodal computed tomography (mCT). We hypothesised that WBV will be associated with unfavourable cerebral perfusion parameters assessed by baseline CT perfusion (CTP) and follow-up diffusion-weighted (DW) magnetic resonance imaging (MRI) volume.

## Materials and methods

### Patients

Consecutive ischemic stroke patients (clinico-radiological diagnosis) presenting in hours (8 am to 5 pm weekdays) at the John Hunter Hospital (Newcastle, Australia) between September 2018 and February 2020 were recruited. All patients underwent acute brain multimodal CT, consisting of brain non-contrast CT (NCCT), CT angiography (CTA) and CTP. When clinically warranted, patients also underwent follow-up brain diffusion-weighted (DWI) magnetic resonance imaging (MRI). Baseline demographics, imaging characteristics and stroke severity assessed using the National Institutes of Health Stroke Scale (NIHSS) were recorded. The study was approved by the Hunter New England Health District human research ethics committee (14/10/15/4.02).

### Imaging

Patients were scanned using a Toshiba Aquilion (Tokyo, Japan) CT scanner. To obtain the perfusion images, a total of 19 acquisitions occurred over 60 s. All perfusion images were post processed using the commercial software MIStar (Apollo Medical Imaging Technology, Melbourne, Australia). Previously validated thresholds were applied in order to measure the volume of the acute perfusion lesion (relative delay time (DT) > 3 s) and acute ischemic core (relative cerebral blood flow – CBF- < 30% within the perfusion lesion) [20, 21]. Penumbral volume was calculated as the volume of the perfusion deficit (DT threshold > 3 s) minus the volume of the ischemic core (relative CBF threshold < 30% within the DT > 3 s lesion). The volume of DT > 6 and > 8 s lesions were also recorded when present. A mismatch ratio, a ratio between the extent of hypoperfused tissue at risk for infarction and the irreversibly damaged infarct core, was calculated by dividing DT3 lesion volume by the infarct core volume. Collateral status was assessed using the multiphase CTA reconstructed from CTP. Collaterals were graded according to the Alberta Stroke Program Early CT Score (ASPECTS) collateral score by a single neurologist [22, 23]. Follow-up DWI was performed (generally within 24 to 48 h) using Avanto 1.5 T or Vario 3 T scanners (Siemens, Munich, Germany).

### WBV measurement

WBV was measured in duplicate within 2 h after sampling in accordance with the new guidelines for hemorheological laboratory techniques [24]. Five millilitres of EDTA blood sample was obtained from the patients prior to any stroke treatment. The measurement was carried out at 37°^**.**^C using a Brookfield DV-II + programmable viscometer (MA, USA), using a CP40 spindle. Whole blood adjusted to 40% hematocrit was used for the WBV measurement. Blood is a non-Newtonian fluid and its viscosity varies with the shear rate. For the present study, viscosity was measured at the shear rate of 20 s^−1^. This shear rate was chosen as it is the lowest possible shear rate that can provide accurate viscosity measures with the viscometer used. The low shear rate viscosity corresponds most closely with the environment in smaller blood vessels.

Viscometer performance was assessed against silicone viscosity standard fluid (Brookfield Amtek). Silicone Standards are Newtonian fluids which are accurate to ± 1% of viscosity value. The inter-assay co-efficient of variation obtained with this standard for WBV measurement was 8.2%.

### Statistical analysis

Statistical analyses were performed with IBM SPSS Statistics 26 software. Descriptive results and quantitative baseline patient characteristics were presented as mean and standard deviation (SD) or median and interquartile range (IQR). Bivariate Pearson’s correlation was performed to examine the crude correlation of WBV with CTP parameters, DWI volume and stroke severity. For the parameters with significant correlation, a multivariate linear regression was used to confirm the associations after adjusting for the occlusion site and collateral status. Statistical significance was set at *P* < 0.05.

## Results

Between September 2018 and February 2020, a total of 512 suspected stroke patients underwent acute CTP. Eighty-two confirmed ischemic stroke patients were recruited. Four hundred thirty patients were not included due to (a) after-hours visit (most of the cases); (b) unconfirmed ischemic stroke diagnosis; (c) not consented or (d) unable to obtain the blood sample before recanalization therapy.

Of the 82 recruited patients, the mean age was 72.7 (12.7) years and 47 (57%) were male. The median baseline NIHSS was 9 (4–17) (Table 1). Thirty-nine patients (47.5%) had a large vessel occlusion in baseline CTA. The most frequent stroke subtype was cardioembolic (*n* = 31, 38%) followed by cryptogenic (*n* = 26, 32%), large-artery atherosclerosis (*n* = 11, 14%), small-vessel occlusion (*n* = 10, 12%) and other determined causes (*n* = 3, 4%). In general, the WBV value was higher in cardioembolic and cryptogenic stroke compared to large-artery atherosclerosis and small-vessel occlusion, but the post hoc analysis did not reach the significant level (Table 2).

**Table 1:** Demographic, clinical and imaging details of patients

Mean age years (mean ± SD)		72.7 ± 12.7
Sex (male)		47 (57%)
Baseline median NIHSS (IQR)		9 (4–17)
WBV centipoise (mean ± SD)		4.92 ± 0.85
Occlusion site (*n*)		
Middle cerebral artery M1		28
Middle cerebral artery M2 or M3		11
Internal carotid artery and/or tandem (ICA/M1)		10
Posterior cerebral artery		2
Common carotid artery		1
Distal (not visible) or no occlusion		30
Clinical history		
Diabetes mellitus (yes/no/missing)		15/60/7
Ischemic heart disease (yes/no/missing)		15/61/6
Hypertension (yes/no/missing)		52/24/6
Dyslipidaemia (yes/no/missing)		25/51/6
Imaging parameters (mL) (median and IQR)		
Perfusion lesion (DT3)		77.5 (40.2–140.0)
Penumbra		64.0 (35.0–105.7)
Core		13.5 (4.7–24.5)
DT6 lesion		28.5 (15.2–71.5)
DT8 lesion		14.0 (6.5–49.5)
Follow-up MRI (*n* = 41) (median and IQR)		
DWI Lesion (*n* = 41)	11.0 (2.5–29)	
Time metrices (hours) (median and IQR)		
Onset* to arrival	2.6 (1.4–10.0)	
Onset to image	3.1 [1.9–12.2]	

^*^Either actual time of onset or time last-seen-well

**Table 2:** Mean comparison of WBV among subtype (TOAST) of acute ischaemic stroke

Aetiology	Large-artery atherosclerosis	Small-vessel occlusion	Cardioembolism	Other determined aetiology	Cryptogenic	*P*-value
*N* (%)	**11 (14%)**	**10 (12%)**	**31 (38%)**	**3 (4%)**	**26 (32%)**	**0.188**
Mean ± SD	**4.88 ± 0.95**	**4.32 ± 0.90**	**5.07 ± 0.58**	**-**	**5.01 ± 1.01**	

The median volume of hypoperfused lesion (DT3 lesion) and core was respectively 77.5 (40.2–140) and 13.5 (4.7–25.5) mL (Table 1). Half of the patients underwent follow-up MRI, with a median follow-up DWI volume of 11.0 (2–27) mL. Out of 82 patients, 7 patients were treated with alteplase alone (thrombolysis), 24 underwent endovascular thrombectomy alone and 6 patients received both therapies. WBV value is missing in 5 out of 82 patients, either due to insufficient blood volume or collection error. Collateral status assessment is missing in 1 out of 82 patients. This is due to lack of dynamic CTA as a result of imaging errors.

In the CTP images, DT3 lesion was seen in 63 patients, DT6 lesion was seen in 48 patients and DT8 lesion was seen in 37 patients. Similarly, 62 patients had ischaemic core lesion in CTP images.

WBV was significantly correlated with CTP parameters: perfusion lesion volume, ischemic core volume and mismatch ratio; DWI volume and baseline NIHSS (Table 3).

**Table 3:** Pearson correlations between WBV and CTP parameters, DWI volume and NIHSS

		DT3 lesion	DT6 lesion	DT8 lesion	Core	MMR	DWI	NIHSS
WBV	*r*	0.390	0.337	0.306	0.392	− 0.365	0.359	0.325
	*P*-value	0.002	0.019	0.066	0.002	0.005	0.025	0.004

In multivariate linear regression models, WBV significantly predicted acute perfusion lesion volume (DT3), core volume and mismatch ratio (MMR) after adjusting for the occlusion site and collateral status (Table 4).

**Table 4:** Multivariable linear regression analysis of WBV on CTP parameters and NIHSS

Dependent variable*	Unstandardised coefficients		Standardised coefficients	*P*-value
	Beta (95% CI)	SE	Beta	
DT3 lesion	20.73 (3.67, 37.79)	8.52	0.219	0.018
DT6 lesion	18.90 (− 0.49, 38.30)	9.61	0.244	0.056
Core	16.61 (2.80, 30.42)	7.71	0.288	0.019
MMR	− 7.74 (− 11.97, − 2.41)	2.38	− 0.391	0.004
NIHSS	0.982 (− 0.41, 2.38)	0.70	0.111	0.167
DWI†	8.315 (− 1.54, 18.17)	4.85	0.247	0.096

^*^All models adjusted for occlusion site and collateral status (ASPECTS)

^†^Adjusted for recanalization status in addition to occlusion site and collateral status

## Discussion

The major finding of the present study was the significant association of WBV with baseline CTP parameters: DT3 lesion, MMR and core. The positive and significant association of WBV with baseline cerebral perfusion lesion (DT3) suggests that elevated WBV accentuates a reduction in blood flow in stroke. The association persists even after adjusting for the effect of collaterals and occlusion site. We observed the negative and significant association of WBV with mismatch ratio, after adjusting for the effect of collaterals and occlusion site. MMR is used in a clinical practice to select ischemic stroke patients for reperfusion therapies during the extended time window [25]. Decreased MMR and larger infarct size have also been shown to predict unfavourable stroke outcome [26]. A mild reduction in cerebral blood flow is normally corrected by local vasodilation (auto-regulation) to maintain cerebral perfusion [27]. Once the capacity of local vasodilation to compensate for a reduction in flow due to occlusion is overwhelmed (failed auto-regulation), the penumbra begins to infarct, after which this tissue is not salvageable despite restoration of cerebral perfusion. At the advent of a vessel occlusion (with corresponding reduction in cerebral blood flow and cerebral blood volume), residual flow via collateral vessels and the microcirculation determines the rate of infarct core growth. Elevated WBV will slow down microcirculation leading to an increased core, thereby decreasing the mismatch ratio, as seen in the present study.

Several previous studies have shown elevated WBV in stroke [6–8, 28–34]. However, whether the elevated WBV reduces cerebral reperfusion in stroke has not been definitely established. The present study demonstrated the independent association of WBV with MMR and perfusion and ischemic lesion volume. The present findings open new possibilities for therapeutic strategies targeting erythrocyte rheology to improve microcirculation. One such therapeutic agent, pentoxifylline, has been trialled in several small studies in the past. Though there are some positive reports on the benefit of this drug [35, 36], there is a lack of consistent evidence regarding its favourable effect on stroke outcome and these studies were conducted before the advent of modern recanalization therapy [37, 38]. Similarly, another potential therapeutic agent arginase inhibitor that improves blood rheology has shown some effect in increasing microvascular flow [39–41]. To verify the favourable outcome of such therapeutic agents that improves WBV and microcirculation, further rigorous clinical trials are warranted.

WBV value was found to be higher in cardioembolic stroke and cryptogenic stroke compared to large-artery atherosclerosis and small-vessel occlusion even though the difference did not reach the level of statistical significance in post hoc analysis. Moreover, there was no significant difference in the WBV between large-artery atherosclerosis and small-vessel occlusion. This is in contrast to the study of Song SH et al. [42], where the WBV in small-vessel occlusion was reported to be significantly higher than in large-artery atherosclerosis and other TOAST subtypes. This difference could be due to the shear rate chosen for the measurement of WBV. Song SH et al. measured WBV at the shear rate of 1 s^−1^. This reflects the shear rate in very small blood vessels (capillaries) aligning more with small-vessel occlusion. We measured WBV at the shear rate of 20 s^−1^. In the present study, WBV was significantly correlated with DWI lesion volume. However, after correcting for the effect of occlusion site, collaterals and recanalization status, the association was not significant. Nevertheless, we only had 41 patients with follow-up DWI lesion volume and 24 of them underwent recanalization therapy. Hence, larger sample size will be required to reach into any conclusion regarding the effect of WBV on infarct growth. Those who received follow-up MRI were more likely to have a lacunar stroke. We had 8 patients with lacunar stroke and all 8 had a follow-up MRI. In addition, patients who suffered very large strokes and were in palliative care after initial work-up typically did not receive follow-up MRI.

Our data showed lower median value of DWI lesion volume compared to CTP perfusion-derived core volume (Table 1). Median core volume analysed only among patient with follow-up DWI was also higher than DWI lesion. We measured core volume using a previously validated threshold in the acute perfusion lesion where acute ischemic core was defined as relative cerebral blood flow (CBF) < 30% compared to the contralateralhemisphere within the perfusion lesion [20, 21]. This threshold for defining core on CTP was established in patients receiving thrombolysis only and is probabilistic, measuring a pattern of blood flow which is incompatible with tissue survival in patients receiving thrombolysis alone. However, sometimes some of that tissue do actually survive, especially in patients who received ECR. In contrast, DWI actually measures dead tissue directly. Thus, Copen et al. suggested that CTP-derived CBV maps cannot reliably substitute for DWI in measuring core volume [43]. This explains why there are a few patients whose DWI lesion is smaller than their CTP core in our study.

The present study showed weak correlation of WBV with baseline NIHSS, but the significance was lost once the relationship was adjusted for occlusion site. No previous work on WBV has reported the association of WBV with stroke outcome measurements: NIHSS or modified Rankin Score. Hence, further study is warranted with larger sample size to consider the effect of WBV on infarct growth and clinical outcomes.

## Limitation

We only had 41 patients with follow-up DWI. The sample size was too small to investigate the association of WBV with infarct growth.

## Conclusion

We have demonstrated a significant correlation between WBV and baseline cerebral lesion volume. The use of CTP is continually growing in acute stroke diagnosis and management. Baseline CTP is routinely performed in stroke centres to increase diagnostic confidence by delineating the penumbra and ischemic core and identifying patients who may benefit from recanalization therapies [44]. Hence, association of WBV with clinically relevant imaging parameters of cerebral blood flow merits further investigation of role of WBV in cerebral haemodynamics of acute ischemic stroke. Lowering WBV pharmaceutically to increase cerebral microcirculation might be an adjunct therapy to boost cerebral reperfusion in acute ischemic stroke.

## Data Availability

Data supporting the findings of this study are available from the corresponding author [PG] on request.
